# Exercise, APOE Genotype, and Testosterone Modulate Gut Microbiome–Cognition Associations in Prostate Cancer Survivors

**DOI:** 10.3390/genes16121507

**Published:** 2025-12-16

**Authors:** Jacob Raber, Abigail O’Niel, Kristin D. Kasschau, Alexandra Pederson, Naomi Robinson, Carolyn Guidarelli, Christopher Chalmers, Kerri Winters-Stone, Thomas J. Sharpton

**Affiliations:** 1 Department of Behavioral Neuroscience, Oregon Health & Science University, Portland, OR 97239, USA; oniela@ohsu.edu (A.O.); alexcpederson@gmail.com (A.P.); robinnao@ohsu.edu (N.R.); 2 Departments of Neurology and Radiation Medicine, Division of Neuroscience, ONPRC, Oregon Health & Science University, Portland, OR 97239, USA; 3 Knight Cancer Institute, Oregon Health and Science University, Portland, OR 97239, USA; borsch@ohsu.edu (C.G.); chalmech@ohsu.edu (C.C.); wintersk@ohsu.edu (K.W.-S.); 4 Department of Microbiology, Oregon State University, Corvallis, OR 97331, USA; kristin.kasschau@oregonstate.edu (K.D.K.); thomas.sharpton@oregonstate.edu (T.J.S.); 5 Division of Oncological Sciences, School of Medicine, Oregon Health and Science University, Portland, OR 97239, USA; 6 Department of Statistics, Oregon State University, Corvallis, OR 97331, USA; 7 Linus Pauling Institute, Oregon State University, Corvallis, OR 97331, USA

**Keywords:** prostate cancer, androgen deprivation therapy, apolipoprotein E, diet, exercise intervention, cognition

## Abstract

**Background:** Men treated with androgen deprivation therapy (ADT) for prostate cancer are at risk for cognitive decline. Patient genetics and endocrine state may shape gut microbiome features that relate to cognition. **Methods:** We studied a subsample of 79 prostate cancer survivors with prior ADT exposure previously enrolled in a randomized controlled exercise trial comparing three training modalities (strength training, Tai Chi training, or stretching control) who completed an additional food-frequency questionnaire and remote Montreal Cognitive Assessment (MoCA) and provided saliva and stool for *APOE* genotyping, salivary testosterone, and 16S rRNA sequencing. We used beta regression for MoCA (scaled 0–1), linear models for testosterone, alpha diversity regressions, PERMANOVA for beta diversity, and DESeq2 for genus-level differential abundance, with false-discovery correction. **Results:** Compared to post-stretching control, post-strength training testing was associated with higher MoCA scores whereas post-Tai Chi testing was not. *APOE* ε4 carriers exhibited a greater testosterone increase with strength training than non-carriers. Testosterone, and its interactions with exercise modality and *APOE* ε2 status, was related to presence/absence-based community structure; *APOE* ε4 interacted with exercise intervention to influence alpha diversity. At the genus level, exercise was linked to lower levels of Bacteroidota taxa (including Muribaculaceae) and higher levels of Enterobacteriaceae; *APOE* ε4 status was linked to higher *Megamonas* and lower Rikenellaceae RC9 levels; and higher salivary testosterone levels were linked to higher Prevotellaceae taxa and *Succinivibrio* levels. Higher MoCA scores were associated with lower abundances of several Firmicutes genera. **Conclusions:** Endocrine state and *APOE* genotype may condition the gut microbiome’s response to exercise intervention in ADT-treated prostate cancer survivors, with downstream associations with cognition. These findings could inform precision survivorship strategies pairing strength training with genotype- and hormone-informed microbiome monitoring to optimize cognitive performance.

## 1. Introduction

The number of cancer survivors continues to increase due to a growing, aging population and improved cancer detection and treatment. Within 10 years, the number of cancer survivors in the US is projected to increase by 31% [1]. Therefore, it is critical to understand how individual genetic susceptibility factors influence symptom burden and the efficacy of mitigation strategies such as exercise. Cancer survivors often experience impaired functioning long after cancer treatment [2]. Often-reported symptoms include behavioral and cognitive changes, including difficulty concentrating, memory impairment, fatigue, and increased anxiety [3]. Cancer survivors are more likely to report a limitation in activities of daily living and an inability to work due to poor health. Symptoms include an impaired ability to think clearly, lack of concentration, memory, depression, loss of sense of self, and reduced physical functioning [4].

Prostate cancer is the most common cancer in men, with cases exceeding 3 million and growing by 100,000 each year in the US alone [5]. While highly survivable, men suffer from the impact of the disease and treatment on their health and well-being [6]. Persistent symptoms and side effects from treatments, including surgery, radiation therapy and androgen ablation, impact physical and cognitive functioning and emotional health.

Assessing genetic factors of neurological vulnerability may increase the understanding of behavioral and cognitive impairments and the efficacy of rehabilitation strategies among prostate cancer patients. One such genetic risk factor is apolipoprotein E (apoE) isoform, which is involved in cholesterol and lipid homeostasis and synaptic functions [7]. The role of apoE in immunomodulation has been recognized [8]. Prostate tumor-derived apoE induces senescence of Triggering Receptor Expressed on Myeloid cells 2 (TREM2)-positive neutrophils. ApoE and TREM2 expression are increased in prostate cancer and correlate with poor prognosis [9]. There are three major isoforms of apoE present in humans: E2, E3, and E4. Compared to E3, E4 is associated with increased risk of developing Alzheimer’s disease (AD) and cognitive impairments might be related to testosterone [10]. Several studies have identified an association between E4 and increased vulnerability to cognitive dysfunction after cancer treatment in survivors with breast cancer, lymphoma, and testicular cancer receiving chemotherapy [11]. ApoE isoforms may also moderate the influence of lifestyle modification and cancer treatment-related toxicities on cognition.

Treatment for prostate cancer often includes androgen deprivation therapy (ADT), but this treatment can increase cardiovascular risk [12] and increase the risk of diabetes and osteoporosis as well [13]. This might be especially a concern in E4 carriers as, in addition to AD risk, E4 carriers have an increased risk of developing cardiovascular disease compared to E3 carriers [14]. Prostate cancer patients on androgen deprivation therapy (ADT) have an increased AD risk compared to men who do not receive ADT, and this is more pronounced when ADT is given for longer than 12 months [15]. While ADT can slow prostate cancer progression, it also causes many side effects that can impact cognitive and physical functioning, and life-style-related factors like exercise might modulate this impact.

Lifestyle-related factors might mediate the relationship between the apoE isoform and long-term cancer-related toxicity. For example, exercise may curb side effects during cancer treatment and lower the risk of cancer recurrence and improve quality of life in survivors [16]. Exercise has cardio-metabolic benefits and may attenuate the increased risk of cardiovascular disease following cancer treatment [17]. Despite recognition of exercise as a salient protective factor against functional decline in E4 carriers in the setting of other medical comorbidities, this relationship has not yet been fully explored in the context of cancer. The *APOE* genotype may modulate long-term cancer-related toxicity through various pathways. E3 can function in an antitumor capacity through suppression of angiogenesis and cell invasion [18]. E2 is associated with a decreased risk of gastric cancer [18]. E4 is associated with prolonged survival in survivors with melanoma; in contrast, E2 is associated with shorter survival [19]. The *APOE* genotype also modulates the physiology in prostate cancer cells by modulating cholesterol metabolism [20]. In a study of fall prevention exercise in post-treatment female cancer survivors aged 50–75 years old (ClinicalTrials.gov NCT01635413), the *APOE* genotype modulated cancer treatment-related side effects and symptoms and response to exercise intervention [21]. Similar effects might be seen in men with prostate cancer.

The gut microbiome interacts with the brain to contribute to health and disease [22]. Gut microbiomes diversify with age, reflect healthy aging, associate with healthy lipids, and predict survival [23]. The gut microbiome communicates with the brain via the gut–brain axis and affects behavioral phenotypes [24,25,26,27,28,29,30,31,32,33]. In mouse studies, sex and genotype modulate the association of the gut microbiome with behavioral and cognitive measures [34,35,36,37]. ApoE is expressed in the gut [38], yields differential inflammation, and impacts gastrointestinal health [39], so it likely influences the structure and function of the gut microbiome. Work in humans and mice [39] support this supposition, finding that microbiome composition varies as a function of *APOE*. For example, in 13-month-old KI mice carrying either E2, E3, or E4, *APOE* genotypes lead to significantly distinct gut microbiome compositions.

In prostate cancer patients who have received ADT, the gut microbiome can generate testosterone and as a result promote prostate cancer growth [40]. The gut microbiome might be critical in prostate cancer in relation to several aspects. In prostate cancer patients on ADT or in castrated mice, the gut microbiome can generate testosterone and fuel prostate cancer growth and in this way contribute to a treatment-resistance condition [41]. There might be long-term effects on the brain too. Given the gut microbiome’s role in mediating cancer [42], hormonal regulation [43], and neurodegenerative disorders [44], variation in the gut microbiome, which can be driven by genetic [39,45] or environmental factors [46,47,48,49,50,51,52,53], might define how ADT impacts these physiological outcomes. ApoE is expressed in the gut, yields differential inflammation, generates testosterone, and impacts gastrointestinal health, so it likely influences the structure and function of the gut microbiome.

In an ancillary study of prostate cancer survivors who recently participated in an exercise trial, we had an opportunity to assess whether the gut microbiome is associated with cognitive measures in men with prostate cancer treated with ADT, and whether the *APOE* genotype, exercise history, and salivary testosterone levels modulate this relationship.

## 2. Materials and Methods

### 2.1. Subject Recruitment

Following consent, we recruited 92 men with a history of androgen deprivation therapy (ADT) for prostate cancer (currently being treated or treated within the last 10 years) who had recently completed participation in a clinical fall prevention exercise trial (men were randomized to strength or Tai Chi training compared to a stretching control group) as part of the GET FIT prostate trial (NCT03741335) [54]. All three study arms were trained concurrently in supervised group exercise classes for 6 months. The study participants are well characterized by self-reports they completed.

### 2.2. Cognitive Testing

The Montreal Cognitive Assessment [55] was administered through videoconferencing (https://www.mocatest.org/remote-moca-testing/) to provide objective cognitive measures (short-term memory, visuospatial ability, executive function, orientation, and attention) in addition to the self-report measures. This test takes only 10 min to administer and cover eight domains.

### 2.3. Diet Questionnaire

Stool donors completed a food frequency questionnaire to provide background information about their diet using the National Cancer Institute DHQIII.

### 2.4. Collection of Saliva and Stool Samples

We collected stool for gut microbiome analysis and saliva samples for *APOE* genotyping and analysis of salivary testosterone (T) levels. The kits for these assays were shipped to the study participants and back so that they did not have to visit OHSU. Saliva samples were stored in a normal −20 °C freezer at home prior to shipping to our laboratory. While salivary testosterone is pretty stable [56], if the samples are not kept cold, bacteria will grow that could interfere with the assay.

### 2.5. 16S rRNA Gut Microbiome Analysis

#### 2.5.1. DNA Extraction and Sequencing

DNA was extracted from stool samples using the DNeasy 96 Powersoil Pro Kit (Qiagen, Hilden, Germany) following the kit protocol. The V4 region of the 16S rRNA gene was amplified using a modified version of the Earth Microbiome Project 16S Illumina (San Diego, CA, USA) Amplicon Protocol [57]. The 515F indexing primers and the 806R primer were used to amplify the V4 region of the small subunit (SSU) rRNA gene, using Platinum II Hot-Start PCR Master Mix (2x) from ThermoFisher Scientific (Waltham, MA, USA). PCR products were visualized on a 1.5% agarose gel to confirm the expected ~350 bp amplicon. Quantification of the PCR amplicons was performed using the Quant-iT 1x ds DNA HS Assay Kit (ThermoFisher Scientific), with fluorescence measured on a BioTek Synergy H1 Hybrid Multi-Mod Plate Reader at the Center for Quantitative Life Sciences (CQLS) at Oregon State University. Amplicons (100 µg per sample) from each stool sample, including molecular-grade water and kit blank quality controls, were pooled to generate a composite 16S library. The pooled library was purified using the QIAquick PCR Purificiation Kit (Qiagen) and quantified using the Qubit 1x dsDNA HS Assay Kit on the Qubit 2.0 fluorometer. Amplicons were sequenced on an Illumina MiSeq (v3; 2 × 300 bp) at the CQLS, targeting ≥40,000 reads per sample.

#### 2.5.2. Sequence Processing

Demultiplexed 16S reads were trimmed and processed with DADA2 [58] for quality control, denoising, paired-end merging, chimera filtering, and amplicon sequence variant (ASV) selection. Taxonomy was assigned using the SILVA database [59], and phylogenies were constructed using FastTree v2.2 [60].

### 2.6. Statistical Analysis

#### 2.6.1. Data Preparation

One sample with an unclear *APOE* genotype was excluded, along with five samples that could not be genotyped for technical reasons, resulting in seventy-nine samples for analysis. *APOE* genotypes were categorized three ways: (1) tripartite classification (E3_E3 homozygotes, E2 carriers, E4 carriers), (2) E4 carrier status (carrier vs. non-carrier), (3) E2 carrier status (carrier vs. non-carrier). Samples with E2/E4 genotypes were excluded from genetic analyses. Testosterone levels were log-transformed to improve normality. Microbiome data were rarefied to an even depth (minimum library size) using the phyloseq package in R.

#### 2.6.2. Host Factor Analysis

To understand relationships between host factors independent of microbiome considerations, we analyzed associations between exercise intervention, APOE genotype, cognitive function, and hormonal status. For cognitive function analysis, MoCA scores were scaled to the 0–1 interval and analyzed using beta regression models. Model selection was performed using the Akaike Information Criterion (AIC), testing main effects and interactions between exercise intervention, APOE status, and testosterone levels. The best model was selected based on the lowest AIC value. For hormonal analysis, log-transformed testosterone levels were analyzed using linear models with APOE status, exercise intervention, and their interaction as predictors. Model selection followed the same AIC-based approach. Direct effects of APOE genotype on MoCA scores and testosterone levels were assessed using beta regression and linear models, respectively, with APOE status as the sole predictor.

#### 2.6.3. Microbiome Analyses

Three alpha diversity metrics were calculated: Shannon diversity (accounting for abundance and evenness), Simpson diversity (emphasizing dominant taxa), and observed species richness (total detected taxa). Associations with host factors were assessed using linear regression models for continuous variables and ANOVA for categorical variables. Interaction terms were included to test for exercise by APOE genotype effects. Models were adjusted for relevant covariates when appropriate. Community composition was assessed using Bray–Curtis (abundance-weighted) and Sorenson (presence/absence) dissimilarity matrices. Distance-based redundancy analysis (dbRDA) as implemented by the capscale function in R was performed for ordination. Permutational multivariate analysis of variance (PERMANOVA) with 999 permutations tested associations between community composition and host factors, including main effects and interactions. For taxon level analyses, ASVs were agglomerated to genus level, and taxa with <5% prevalence were filtered, resulting in 211 genera for analysis. Differential abundance testing was performed using DESeq2 with negative binomial generalized linear models. Separate models were fit for each host factor (exercise intervention, APOE genotype, testosterone levels, MoCA scores, and age). False-discovery rate correction was applied to account of multiple tests using the Benjamini–Hochberg approach.

## 3. Results

### 3.1. Participant Characteristics and Data Quality

To establish the foundation for our analyses, we first characterized the study cohort and assessed data completeness. The final analytical cohort consisted of 79 men with prostate cancer who had received ADT, with 47 (59.5%) having metastatic disease. Among this subsample from the full trial, men were assigned to one of three study arms: strength training (*n* = 36, 45.6%), Tai Chi (*n* = 23, 29.1%), and stretching control (*n* = 20, 25.3%). Data completeness exceeded 88% for all key variables. MoCA cognitive scores were available for 78/79 samples (98.7%), ranging from 11 to 21 (median = 17, mean = 16.73, SD = 2.4), with scores below 15 in 14 participants indicating moderate cognitive impairment. Testosterone measurements, available for 73/79 samples (92.4%), showed substantial variation after log-transformation (range: 0.97–5.24, median = 4.00, mean = 3.83, SD = 0.87). Ages ranged from 55 to 84 years (mean = 71.2, SD = 6.8), with complete data for all participants. *APOE* genotyping successfully characterized 70/79 samples (88.6%), revealing a distribution consistent with general population frequencies: 46 E3/E3 homozygotes (65.7%), 17 E4 carriers (24.3%), and 7 E2 carriers (10.0%). The high data completeness and balanced group distributions provided robust statistical power for downstream analyses.

### 3.2. Host Factor Relationships Independent of Microbiome

**Figure 1:**
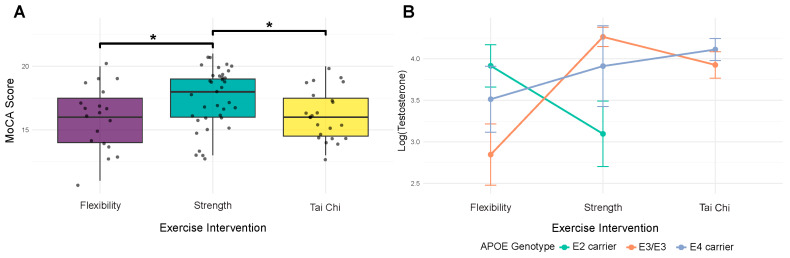
Strength training participation is associated with better cognitive function and may moderate testosterone responses in ApoE4 carriers. (**A**) MoCA cognitive scores by exercise modality. Participation in strength training was associated with higher MoCA scores than participation in flexibility (beta = 0.794, *p* = 0.007) and Tai Chi (beta = 0.617, *p* = 0.018) exercise. Tai Chi does not differ from flexibility (beta = 0.212, *p* = 0.479). Boxplots show group distributions; brackets with asterisks indicate significant pairwise differences using *p* < 0.05 as a threshold. (**B**) Testosterone varies as a function of the interaction between ApoE and exercise. We observe a significant interaction (*p* = 0.023) such that E4 carriers exhibit a positive association of strength training participation on testosterone (beta = 0.847, *p* = 0.012), whereas E2 (likely due to small sample size) and E3_E3 groups show no significant association with exercise modality. Lines and points summarize group means with standard errors.

Before examining microbiome associations, we analyzed relationships between host factors to understand their interdependencies. For cognitive function, AIC-based model selection identified the combination of exercise modality and testosterone as the best predictor of MoCA scores (AIC = −3.34, pseudo *R*^2^ = 0.127). Strength training showed a significant positive relationship with cognitive function (β = 0.794, *p* = 0.007), while Tai Chi showed no significant difference to flexibility control (Figure 1A). Testosterone demonstrated a positive but non-significant association with MoCA scores (β = 0.183, *p* = 0.163) (Figure 1B).

Analysis of hormonal function revealed a significant interaction between *APOE* genotype and exercise modality on testosterone levels (interaction *p* = 0.023, model *R*^2^ = 0.156). This interaction was driven by E4 carriers, who showed a significant positive association with participation in strength training (β = 0.847, *p* = 0.012), while E3/E3 homozygotes and E2 carriers showed no significant association with any exercise modality. Direct examination of *APOE* effects revealed no significant associations with either MoCA scores (pseudo *R*^2^ = 0.007, all *p* > 0.63) or testosterone levels (*R*^2^ = 0.012, all *p* > 0.21), indicating that genetic effects operate primarily through interactions rather than direct pathways.

### 3.3. Alpha Diversity Patterns Reveal Complex Host–Microbiome Interactions

**Figure 2:**
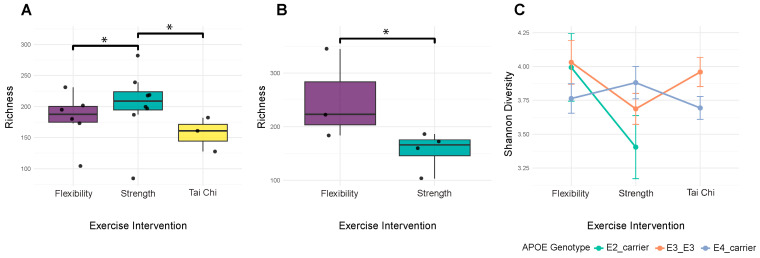
*APOE* genotype modifies exercise associations with microbiome diversity. (**A**) Observed richness varies by exercise in E4 carriers. This plot only shows data for E4 carrier samples. Strength impacts community richness among E4 carriers (beta = 127.142, *p* = 0.017); brackets mark significant pairwise comparisons with asterisks, which indicate *p* < 0.05. (**B**) Observed species richness varies by exercise among E2 carriers. This plot only shows data for E2 carrier samples. A significant exercise interaction is observed (beta = 105.885, *p* = 0.031); the bracket indicates the significant flexibility vs. strength comparison with the asterisk indicating *p* < 0.05. No Tai Chi group is shown in this plot because our study included no E2 carriers within the Tai Chi group. (**C**) Shannon diversity varies by exercise across ApoE genotypes. The exercise x *APOE* interaction is significant (beta = 0.857, *p* = 0.047); mean Shannon diversity ± standard error lines by genotype illustrate genotype-specific exercise responses.

**Figure 3:**
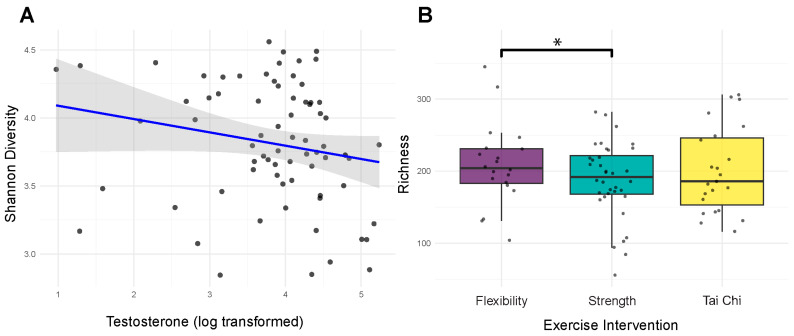
Testosterone and exercise modality are independently associated with gut microbiome diversity. (**A**) Shannon diversity vs. testosterone (on log scale) as illustrated by a scatterplot with linear fit reveals a negative association (Spearman rho ≈ −0.232, *p* ≈ 0.049). (**B**) Observed species richness by exercise intervention. Boxplots show distributions across groups. A bracket highlights the significant strength main effect relative to flexibility (beta ≈ −98.8, *p* ≈ 0.023); the asterisk denotes statistical significance using *p* < 0.05.

Alpha diversity metrics captured distinct aspects of microbial community structure. Shannon diversity (mean: 3.799 ± 0.457) and Simpson diversity (mean: 0.940 ± 0.041) reflected community evenness, while observed species richness (mean: 196.9 ± 55.6) measured community complexity. The wide ranges observed across all metrics indicated substantial inter-individual variation in microbiome composition. Age significantly influenced Simpson diversity (*F* = 1.79, *p* = 0.035, *R*^2^ = 0.515), with trending effects on other diversity metrics (observed species (*F* = 1.49, *p* = 0.106, *R*^2^ = 0.469) and Shannon diversity (*F* = 1.46, *p* = 0.121, *R*^2^ = 0.463)), suggesting age-related changes affect overall community structure rather than specific diversity components. The relationship between exercise modality and microbiome diversity depended critically on *APOE* genotype. For E4 carriers, exercise modality produced significant positive interactions with observed species richness (β = 127.142, *p* = 0.017) (Figure 2A) and Shannon diversity (β = 0.857, *p* = 0.047) (Figure 2C), while the main association with strength training participation was negative (β = −98.837, *p* = 0.023) (Figure 2B). E2 carriers showed a different pattern, with significant exercise intervention interactions only for observed species (β = 105.885, *p* = 0.031) (Figure 2B). These genotype-specific responses suggest that genetic background could modulate the microbiome’s response to exercise. Hormonal status also influenced diversity patterns. Salivary testosterone levels showed significant negative correlations with Shannon diversity (ρ = −0.232, *p* = 0.049) (Figure 3A) and trending negative association with Simpson diversity (ρ = −0.195, *p* = 0.099). MoCA scores demonstrated consistent positive trends across all diversity metrics (*p* = 0.060–0.099), though none reached significance after correction. Together, these findings indicate that microbiome diversity in prostate cancer patients reflects a complex interplay of demographic, genetic, hormonal, and behavioral factors.

### 3.4. Beta Diversity Analysis Reveals Hormonal and Interactive Effects on Community Composition

**Figure 4:**
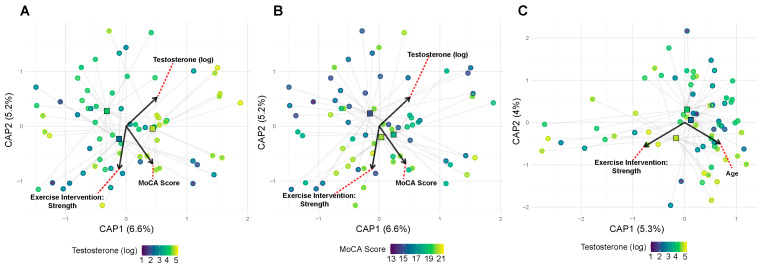
Testosterone and cognitive function structure gut microbial community composition. (**A**) Community structure by testosterone (Sorenson). Testosterone shows a significant main effect (*R*^2^ = 0.024, *p* = 0.017). Points are filled by log-transformed testosterone levels. To aid interpretation for a continuous covariate, samples are split into tertiles (low/mid/high); faint “spider” segments connect points to their tertile centroid, and larger squares show centroids filled by the tertile midpoint on the same color scale. A thin colored ring around each point indicates tertile membership. Covariate vectors indicate the strongest drivers of ordination axes, where red dotted lines connect covariate vector labels to their corresponding vectors. (**B**) Community structure by cognitive function (Sorenson). MoCA shows a suggestive association (*R*^2^ = 0.020, *p* = 0.056). Same styling as A except with MoCA as the continuous fill; centroids are filled by MoCA tertile midpoints. (**C**) Community structure by testosterone (Bray–Curtis). Testosterone shows a suggestive association (*R*^2^ = 0.019, *p* = 0.103). Same styling as A except with Bray–Curtis distances and testosterone as the continuous fill.

Community composition analysis provided insights that were complementary to alpha diversity findings. Unlike diversity metrics, which showed strong age effects, community composition was primarily influenced by hormonal factors. Testosterone levels significantly affected bacterial community membership (Sorenson distances: *R*^2^ = 0.024, *p* = 0.017) (Figure 4A), with trending effects on abundance-weighted metrics (Bray–Curtis: R^2^ = 0.019, *p* = 0.103) (Figure 4C). Neither exercise intervention nor *APOE* status showed significant main effects on community structure (all *p* > 0.15). MoCA showed a suggestive association with community composition (*R*^2^ = 0.020, *p* = 0.056) (Figure 4B).

**Figure 5:**
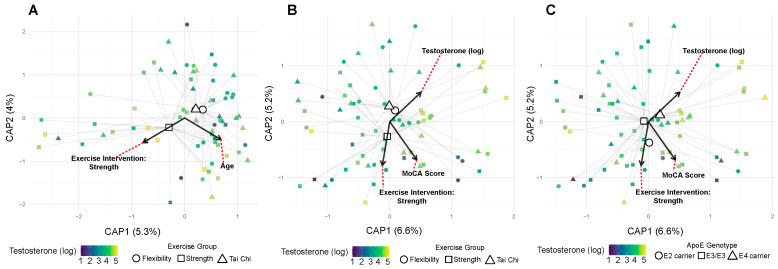
Exercise and *APOE* genotype interact with testosterone to shape microbial community structure. (**A**) Community structure by exercise and testosterone (Bray–Curtis). The exercise-by-testosterone interaction is significant (*R*^2^ = 0.037, *p* = 0.049). Points are filled by log-transformed testosterone levels and shaped by exercise modality (circle/square/triangle). Faint spider segments connect points to their exercise-modality centroids; centroids are drawn as larger versions of the same shape. Covariate vectors indicate the strongest drivers of ordination axes, where red dotted lines connect covariate vector labels to their corresponding vectors. (**B**) Community structure by exercise and testosterone (Sorenson). The exercise-by-testosterone interaction is significant (*R*^2^ = 0.041, *p* = 0.012). Same styling as in (**A**). (**C**) Community structure by *APOE* genotype and testosterone (Sorenson). The *APOE*-by-testosterone interaction is significant (*R*^2^ = 0.022, *p* = 0.046). Same styling as in A with *APOE* genotype shaping instead of the exercise group.

However, significant interactions emerged when examining combined factors. Exercise and testosterone showed synergistic effects on both abundance-based (Bray–Curtis: *R*^2^ = 0.037, *p* = 0.049) and membership-based (Sorenson: *R*^2^ = 0.041, *p* = 0.012) (Figure 5A) community metrics. Additionally, E2 carrier status modified the testosterone effect on community membership (Sorenson: *R*^2^ = 0.022, *p* = 0.046). MoCA scores showed a trending association with community composition (Sorenson: *R*^2^ = 0.020, *p* = 0.056) (Figure 5B), and salivary testosterone levels showed a suggestive association (*R*^2^ = 0.019, *p* = 0.103) (Figure 5C). These beta diversity patterns suggest that while individual factors may have limited effects on overall community structure, their interactions create distinct microbial signatures. The stronger effects on Sorenson (presence/absence) versus Bray–Curtis (abundance-weighted) distances indicate that hormonal and exercise factors primarily affect which bacteria are present rather than their relative abundances.

### 3.5. Taxon-Level Analysis Identifies Specific Bacterial Signatures of Host Factors

**Figure 6:**
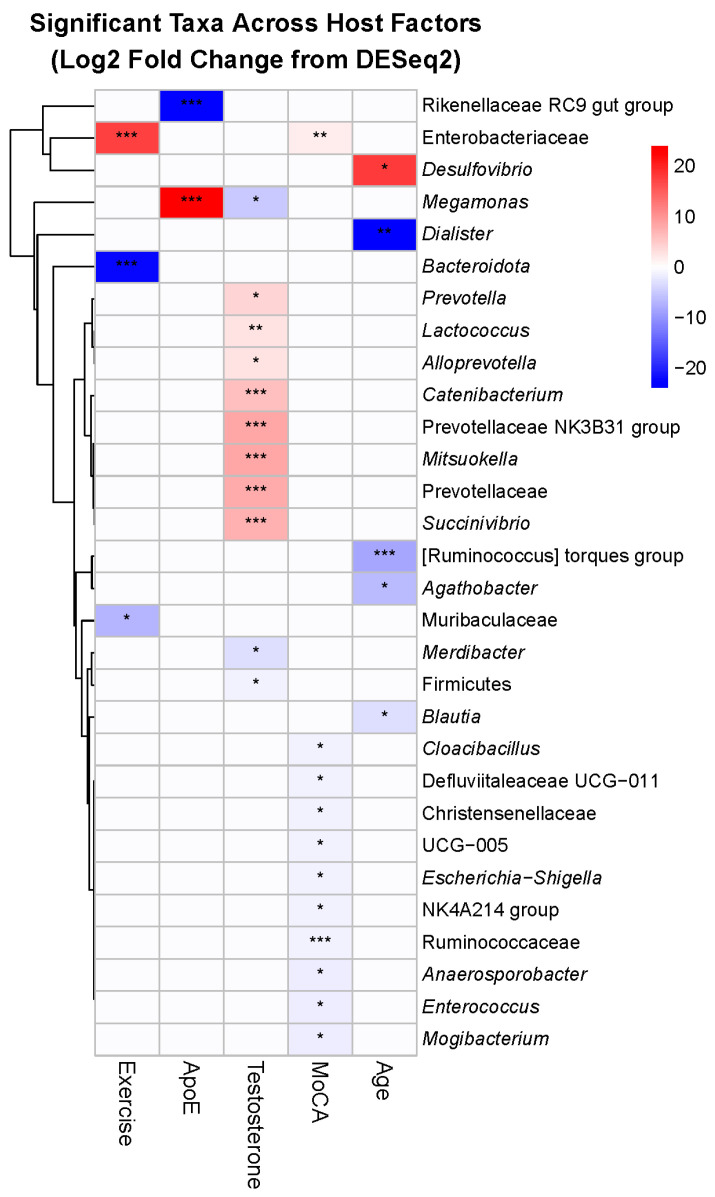
Host factors associate with distinct bacterial taxon relative abundances. Significant taxa (FDR-corrected *p* < 0.05) were identified across host factors: exercise (3 taxa), ApoE genotype (2 taxa), testosterone (11 taxa), MoCA score (11 taxa), and age (5 taxa). Rows represent significant taxa (genus-level); columns are host factors. Values are DESeq2 log2 fold changes; cells display significance with asterisks (* *p* < 0.05, ** *p* < 0.01, *** *p* < 0.001). To improve legibility, very small but significant effects are visualized with a minimum color intensity so that all significant cells appear non-white, while non-significant effects remain white (0). Row labels prioritize genus (or else family or phylum when genus annotations are not available) for readability; color breaks are symmetric about zero.

To identify the specific bacteria driving diversity patterns, we analyzed differential abundance across 211 genus-level taxa, revealing 32 significant associations (15.2%) with host factors, indicating selective rather than global microbiome changes (Figure 6). Exercise modality produced contrasting effects on anaerobic bacteria. Two obligate anaerobes decreased significantly: an unclassified Bacteroidota taxon (log2FC = −21.96, padj = 1.23 × 10^−15^) and Muribaculaceae (log2FC = −6.80, padj = 0.028). Conversely, the facultative anaerobe family Enterobacteriaceae increased markedly (log2FC = 17.61, padj = 8.93 × 10^−6^). This shift from obligate to facultative anaerobes may reflect exercise-induced changes in gut oxygen tension or transit time.

*APOE* E4 carrier status was associated with a phylum-level trade-off: increased Megamonas from Firmicutes (log2FC = 23.86, padj = 4.26 × 10^−10^) coupled with decreased Rikenellaceae RC9 from Bacteroidota (log2FC = −23.36, padj = 2.88 × 10^−11^). This Firmicutes/Bacteroidota ratio shift represents a fundamental alteration in community structure associated with the E4 genotype.

Salivary testosterone levels showed the broadest taxonomic associations, affecting 11 genera across multiple phyla. The Prevotellaceae family showed consistent positive associations with testosterone, including the NK3B31 group (log2FC = 8.30, *padj* = 5.74 × 10^−6^), Prevotella (log2FC = 4.14, *padj* = 0.025), and Alloprevotella (log2FC = 2.84, *padj* = 0.0014). The proteobacterium Succinivibrio also increased (log2FC = 7.40, *padj* = 1.01 × 10^−6^), while Catenibacterium decreased (log2FC = −5.59, *padj* = 0.00012). Given that some gut bacteria can metabolize hormones, these associations may reflect bidirectional host–microbe interactions.

Cognitive function showed intriguing taxonomic patterns, with 11 associated genera predominantly from Firmicutes (8/11). Most Firmicutes members decreased with higher MoCA scores, including multiple Ruminococcaceae genera (UCG-005: log2FC = −0.46, *padj* = 0.012; NK4A214: log2FC = −0.42, *padj* = 0.033). In contrast, Enterobacteriaceae increased with better cognitive function (log2FC = 1.89, *padj* = 0.0056), paralleling its positive association with exercise.

Age uniformly reduced putatively beneficial taxa, particularly within the Lachnospiraceae family. Three members showed significant age-related decreases: [Ruminococcus] torques (log2FC = −8.24, *padj* = 0.00053), Agathobacter (log2FC = −6.47, *padj* = 0.036), and Blautia (log2FC = −2.91, *padj* = 0.036). Dialister showed the largest magnitude decrease (log2FC = −23.85, *padj* = 0.0032). These taxa are known short-chain fatty acid producers, suggesting an age-related loss of beneficial metabolic functions. Overall, the taxon-level findings provide mechanistic insights into the diversity patterns, revealing that host factors do not uniformly affect the microbiome but rather target specific bacterial groups with distinct functional capabilities.

## 4. Discussion

In this cohort of men with prostate cancer and prior or current androgen deprivation therapy (ADT), we observed three principal findings. First, host factors independent of the microbiome showed that participation in strength training was associated with higher Montreal Cognitive Assessment (MoCA) scores, and *APOE* genotype modified the association of exercise with salivary testosterone, with E4 carriers that received strength training demonstrating higher testosterone levels. Second, gut community structure was associated with hormonal status—salivary testosterone levels related to differences in presence/absence-based community metrics—and with interactions between salivary testosterone and exercise modality (and, for membership, testosterone and *APOE* E2 status). Third, taxon-level analyses identified specific bacterial signatures linked to exercise modality (decreased Bacteroidota taxa and increased Enterobacteriaceae), *APOE* E4 (increased *Megamonas*, decreased Rikenellaceae RC9), salivary testosterone (increased Prevotellaceae taxa and *Succinivibrio*), age (decreases in several Lachnospiraceae), and cognitive scores (multiple Firmicutes genera decreased as MoCA increased). Together, these findings support a model in which genetic background and endocrine milieu shape the microbiome’s association with exercise history, with potential implications for cognition in ADT-treated prostate cancer survivors.

The gut–brain axis is increasingly recognized as a determinant of behavioral and cognitive phenotypes [24,31,32]. In our study, testosterone showed a main association with community membership (Sorenson) and interacted with exercise history to influence both membership and abundance-weighted metrics, suggesting that endocrine state could be a key dimension along which exercise “reprograms” gut communities after ADT. These associations, while not evidence of causal relationships themselves, are consistent with evidence for bidirectional links between sex steroids and the microbiome [61] and with mechanistic studies showing that commensal bacteria can synthesize androgens that fuel endocrine resistance in prostate cancer [41]. Notably, *APOE* E2 status modified testosterone’s association with membership, and *APOE* E4 status interacted with exercise to influence alpha diversity (observed richness and Shannon). Prior human and murine work have shown that the *APOE* genotype shapes gut microbial composition and function [39,45], providing a biologically plausible basis for the genotype-dependent microbiome responses we detected.

Age was a prominent correlate of alpha diversity, and age-related declines were evident in several genera within Lachnospiraceae and in *Dialister*. This is consistent with population studies linking aging to characteristic shifts in gut communities that track health status and survival [23]. The observed associations between MoCA and community membership (trend) and between MoCA and multiple genera (notably several Firmicutes with lower abundance at higher MoCA) further underscore cognitive–microbiome coupling in this clinical context, while also highlighting that directionality may differ from patterns described in community-dwelling older adults, likely reflecting disease, treatment (including ADT), and comorbidity influences [24,31,32].

Exercise history was associated with reduced abundance of two Bacteroidota taxa (including Muribaculaceae) and higher Enterobacteriaceae. The *APOE* E4-linked profile—higher *Megamonas* and lower Rikenellaceae RC9—represents a shift across Firmicutes/Bacteroidota boundaries and mirrors prior reports of *APOE*-dependent restructuring of the gut ecosystem [39,45]. Salivary testosterone levels correlated positively with multiple Prevotellaceae taxa and *Succinivibrio*, genera frequently linked to carbohydrate fermentation and, in some settings, to host metabolic and hormonal phenotypes [61]. Although our cross-sectional design precludes causal inference, these signals are consistent with a scenario in which endocrine status, exercise behavior, and genetic background jointly select for microbial modules with distinct metabolic capacities.

From a clinical perspective, three implications emerge. First, recent participation in strength training was associated with better cognitive performance independent of *APOE* genotype and microbiome measures, reinforcing current guidance to include strength training in survivorship care plans [16]. Second, the *APOE* E4-specific testosterone response to strength training raises the possibility that *APOE* genotype-informed exercise prescriptions [21] could be leveraged to optimize endocrine and functional outcomes after ADT. Third, given evidence that gut microbes can contribute to extra-gonadal androgen biosynthesis, and thereby to ADT resistance [41], the observed testosterone–microbiome coupling suggests that tracking (and potentially targeting) gut community features may complement exercise-based strategies to improve long-term neurological and oncologic outcomes in patients with prostate cancer.

This study has several limitations. The analysis is cross-sectional and not longitudinal. The microbiome data are taxonomic data only and do not include metagenomic or metabolomic data to determine pathway changes related to androgen or short-chain fatty acid metabolism. The cohort is relatively small and based on the occurrence of *APOE* ε2 (8%), ε3 (77%), and ε4 (15%); alleles in the general population contains much less E2 and E4 than E3 carriers. Cognitive outcomes were only assessed with the MoCA. It is conceivable that as a result, subtle or domain-specific deficits in men with prostate cancer treated with ADT might have been missed. Diet was recorded but not modeled in depth as a potential confounder.

Going forward, prospective, longitudinal trials that integrate *APOE* genotyping, detailed ADT exposure (drug, dose, duration), standardized strength-training protocols, and multi-omics (shotgun metagenomics, metabolomics, including steroidomics and short-chain fatty acids) are needed to test whether targeted exercise alters microbial functions tied to androgen biology and cognition [41,49,51,52]. Parallel measurement of neuroimmune markers and microglial-modulating pathways relevant to *APOE* genotype and *TREM2* [8] could clarify mechanistic links between exercise, the microbiome, and neurocognitive outcomes. In addition, it will be important to assess whether in men with prostate cancer who received ADT and exercise intervention there might be signatures in the diet or diet-based scores that affect the gut microbiome and/or cognition.

In summary, our results indicate that genetic background and endocrine milieu potentially condition the gut microbiome’s response to exercise in ADT-treated prostate cancer survivors, with downstream associations with cognition. These data may inform precision survivorship strategies that pair strength training with *APOE* genotype- and hormone-informed monitoring of the gut ecosystem to optimize cognitive function.

## Data Availability

The original contributions presented in this study are included in the article. Further inquiries can be directed to the corresponding author.
